# Induction of endoplasmic reticulum calcium pump expression during early leukemic B cell differentiation

**DOI:** 10.1186/s13046-017-0556-7

**Published:** 2017-06-26

**Authors:** Lamia Aït Ghezali, Atousa Arbabian, Hervé Roudot, Jean-Philippe Brouland, Fanny Baran-Marszak, Evelyn Salvaris, Andrew Boyd, Hans G. Drexler, Agnes Enyedi, Remi Letestu, Nadine Varin-Blank, Bela Papp

**Affiliations:** 1 Institut National de la Santé et de la Recherche Médicale, U978 Bobigny, France; 2 Université Paris-13, PRES Sorbonne Paris-Cité, 74, rue Marcel Cachin 93017, Bobigny, France; 3 Département de Virologie, Institut Pasteur, Paris, France; 4 Service d’Anatomie et Cytologie Pathologiques, Hôpital Lariboisière, Paris, France; 5 Service d’Hématologie Biologique, Hôpitaux Universitaires Paris Seine-Saint-Denis, AP-HP, Hôpital Avicenne, Bobigny, France; 6 Immunology Research Centre, St Vincent’s Hospital, Melbourne, VIC Australia; 7 Department of Medicine, University of Queensland, Queensland, Australia; 8 Deutsche Sammlung von Mikroorganismen und Zellkulturen GmbH, Brauschweig, Germany; 9 Second Institute of Pathology, Semmelweis University Medical School, Budapest, Hungary; 10 U978 Inserm, UFR SMBH, Université Paris-13, 74, rue Marcel Cachin, 93017 Bobigny, France

**Keywords:** Pre-B acute lymphoblastic leukemia, Phorbol ester, Endoplasmic reticulum, SERCA, Calcium transport

## Abstract

**Background:**

Endoplasmic reticulum (ER) calcium storage and release play important roles in B lymphocyte maturation, survival, antigen-dependent cell activation and immunoglobulin synthesis. Calcium is accumulated in the endoplasmic reticulum (ER) by Sarco/Endoplasmic Reticulum Calcium ATPases (SERCA enzymes). Because lymphocyte function is critically dependent on SERCA activity, it is important to understand qualitative and quantitative changes of SERCA protein expression that occur during B lymphoid differentiation and leukemogenesis.

**Methods:**

In this work we investigated the modulation of SERCA expression during the pharmacologically induced differentiation of leukemic precursor B lymphoblast cell lines that carry the E2A-PBX1 fusion oncoprotein. Changes of SERCA levels during differentiation were determined and compared to those of established early B lymphoid differentiation markers. SERCA expression of the cells was compared to that of mature B cell lines as well, and the effect of the direct inhibition of SERCA-dependent calcium transport on the differentiation process was investigated.

**Results:**

We show that E2A-PBX1^+^ leukemia cells simultaneously express SERCA2 and SERCA3-type calcium pumps; however, their SERCA3 expression is markedly inferior to that of mature B cells. Activation of protein kinase C enzymes by phorbol ester leads to phenotypic differentiation of the cells, and this is accompanied by the induction of SERCA3 expression. Direct pharmacological inhibition of SERCA-dependent calcium transport during phorbol ester treatment interferes with the differentiation process.

**Conclusion:**

These data show that the calcium pump composition of the ER is concurrent with increased SERCA3 expression during the differentiation of precursor B acute lymphoblastic leukemia cells, that a cross-talk exists between SERCA function and the control of differentiation, and that SERCA3 may constitute an interesting new marker for the study of early B cell phenotype.

**Electronic supplementary material:**

The online version of this article (doi:10.1186/s13046-017-0556-7) contains supplementary material, which is available to authorized users.

## Background

Calcium signaling is essential for B lymphocyte responses. The activation of the B cell antigen receptor and other important receptors leads, via PLC-γ activation and subsequent IP3 production, to the release of calcium accumulated in the ER via IP3 receptor calcium channels. ER calcium release, coupled to capacitative calcium influx from the extracellular space through Orai-type calcium channels leads to increased cytosolic calcium levels, the activation of calcineurin and various PKC isoforms and the activation of NF-κB and NFAT-type transcription factors that orchestrate B cell activation [1–10].

Calcium-dependent activation is critically dependent on the function of Sarco/Endoplasmic Reticulum Calcium ATPases (SERCA enzymes). Located in the ER membrane, SERCA proteins accumulate calcium ions from the cytosol into the ER lumen by ATP-dependent active transport, creating a several thousand fold calcium concentration gradient from the ER (high micromolar) towards the cytosol (low nanomolar) [11–14]. SERCA activity is therefore a prerequisite for IP3-induced calcium release to occur [15, 16], and, by recapturing calcium during a calcium release event, SERCA enzymes also shape the amplitude and the duration of calcium signals [17], and thus modulate cell activation [18]. Mature B cells express simultaneously SERCA2b and SERCA3-type calcium pumps [19, 20]. The calcium affinity of SERCA3 is markedly weaker (K_Ca_^2 +^ ~1.2 μM) than that of SERCA2b (K_Ca_^2 +^ ~0.2 μM) [13, 21]. Due to its lower calcium affinity, SERCA3 will tolerate higher calcium rises during ER calcium release [13, 21–24]. The SERCA2b/SERCA3 protein ratio constitutes a unique mechanism whereby the avidity of the ER for calcium can be modulated in a cell [25, 26].

Early B cell differentiation proceeds through several consecutive steps during which an initially immature lymphoid precursor acquires the cellular machinery required for the function of the differentiated B cell. Phenotypic changes that occur during this process, such as the loss or gain of various markers and gene expression regulatory networks, have already been described in considerable detail [27–29]. However, little is known so far about the functional remodeling of ER calcium homeostasis and about changes of SERCA-dependent ER calcium sequestration that may occur during early B lymphoid differentiation.

Precursor-B ALL cells are blocked at early stages of B-cell differentiation due to the presence of various genetic lesions that give rise to different fusion oncoproteins such as E2A-PBX1 (TCF3-PBX1), BCR-ABL1, MLL-ENL (KMT2A-MLLT1), TEL-AML1 (ETV6-RUNX1) or others. These oncoproteins activate distinct molecular oncogenic mechanisms, influence prognosis, and are widely used for the molecular classification of precursor-B ALL [30, 31].

In order to investigate SERCA expression during early B cell differentiation, and to gain insight into its defects in leukemia, we investigated the effects of protein kinase C activation by a phorbol ester on the phenotype and SERCA expression of various precursor B ALL cell lines in vitro. Phorbol esters activate PKC [32], and are known to induce differentiation of several myeloid leukemia cell types accompanied by increased SERCA3 expression [33, 34]. We show that the differentiation blockage of E2A-PBX1-expressing precursor B-ALL cells can be abolished in vitro by the pharmacological activation of PKC, and that the differentiation of these cells is accompanied by marked changes in SERCA expression. Whereas untreated cells express simultaneously SERCA2 and SERCA3-type calcium pumps, the expression of SERCA3, a lower calcium affinity isoenzyme [13, 21, 24] is preferentially induced upon differentiation, indicating that ER calcium accumulation is functionally remodeled during this process. In addition, we show that the direct pharmacological inhibition of SERCA-dependent calcium transport interferes with the differentiation process as detected by CD20 expression.

## Methods

### Cell culture

Cell lines used in this study were obtained from DSMZ (Braunschweig, Germany), ATCC (Manassas, VA), ECACC (Porton Down, UK) or from the originators (see Additional file 1: Table S1). Authenticity check of cell lines was performed by short tandem repeat analysis by the commercial providers. Early passage cells were cultured in suspension in the appropriate culture medium at 37 °C in humidified cell culture incubators in a 95% air 5% CO_2_ atmosphere. Fetal calf serum that supported vigorous growth of the Kasumi-2 line as single cells without clumping was selected for culture. Mycoplasma screening was performed by polymerase chain reaction using the G238 mycoplasma detection kit of Applied Biological Materials, Euromedex, Souffelweyersheim, France. Cell densities and viabilities of exponentially growing cultures were determined by cell counting using a Malassez chamber and trypan blue exclusion.

### Reagents and treatments

Before treatments cells were centrifuged and suspended in new complete medium at densities indicated in Figures and distributed into 60 cm^2^ plastic Petri dishes. Non-adherent dishes were used for the various cell lines because treatment in adherent conditions resulted in the adherence of a minor fraction (<5%) of the cells following treatments, for example for Kasumi-2 cells. Treatment of MHH-CALL3 cells was done in cell culture grade adherent Petri dishes, since treatment of this cell line leads to the adherence of the overwhelming majority (>90%) of viable cells.

PMA, Gö 6983 and GF 109203X, thapsigargin, cyclopiazonic acid and 2,5-di(*tert*)butyl-1,4-benzohidroquinone were obtained from Sigma-Aldrich France, Saint-Quentin Fallavier, France. Drugs were added to cells from concentrated stock solutions made in dimethyl-sulfoxide (DMSO). DMSO vehicle did not exceed 0.01%, was included in controls and did not interfere with the experiments.

When PMA treatments were applied in the presence of Gö 6983 or GF 109203X, these were added to the cell cultures 30 min before the addition of PMA. PMA and thapsigargin in double treatment experiments were added simultaneously. Drugs were thereafter present during the entire experiment.

### Western blotting and flow cytometry

After the appropriate treatments, cells were harvested by centrifugation and washed with ice cold 150 mM NaCl. Total cellular protein was then precipitated with 5% trichloroacetic acid (TCA), centrifuged and dissolved in modified SDS PAGE lysis buffer. This sample preparation process, as well as quantification of the protein content of lysates was performed as described in [35]. Equal amounts of total cellular protein (60 μg/well) were applied to polyacrylamide gels, and SDS PAGE followed by immunoblotting onto nitrocellulose was done as previously described [35]. After electrotransfer, identical protein loading was checked by Ponceau red staining and densitometry [35].

Western blotting for SERCA2 and SERCA3 was performed using the IID8 (Sigma-Aldrich, 0.4 μg/ml) and the PLIM430 mouse monoclonal (10x diluted hybridoma cell culture supernatant) antibodies, respectively, and peroxidase-conjugated anti-mouse Ig antibodies as in [35]. Luminescent signal obtained with the ECL Prime (Amersham) and SuperSignal West Femto Maximum Sensitivity (Thermo Fisher) reagents was detected, quantified and analyzed using a BioRad ChemiDoc MP image acquisition system and the Image Lab 4.0 software. Immunoblot signal intensities were normalized to the corresponding total protein values as obtained by Ponceau red staining, and are expressed as fold change in treated cells *versus* untreated control.

Detection by Western blotting of CD20 (clone L26 purified mouse monoclonal anti-human CD20cy, Dako Denmark A/S, 0.2 μg/ml), RAG-1 (Santa Cruz Biotechnology, sc-5599, H-300, rabbit polyclonal IgG 0.2 μg/ml), TdT (clone EPR2976Y, rabbit hybridoma culture supernatant monoclonal antibody, dilution:3500x, Epitomics), CD19 (clone LE-CD19 purified mouse monoclonal anti-human CD19, Thermo Fisher Scientific, 0.33 μg/ml) was performed similarly.

Detection and analysis of expression of various lymphoid phenotypic markers (CD3, CD5, CD10, CD19, CD20, CD22, CD34, CD38, CD45, FMC7, TdT, κ and λ light chains and IgM) by flow cytometry was done as previously described [36, 37].

### Cytology and immunocytochemistry

Immunocytochemical staining for CD20 expression was performed on cytologic smears. Suspensions of treated and untreated control cells of packed cell volume ratio of approximately 50% were applied to poly-lysine coated microscopic slides and air-dried overnight. Following fixation in acetone at room temperature for 10 min and drying the slides were rehydrated and labeled for CD20 expression using the Clone L26 monoclonal mouse anti-CD20 antibody (Dakocytomation, Les Ulis, France) at a concentration of 6 μg/ml in Dako REAL^TM^ antibody diluent (Dakocytomation), using an indirect avidin-biotin-peroxidase method with 3,3’diaminobenzidine (DAB) as chromogen on an automated immunostainer (Benchmark®, Ventana Medical Systems, Illkirch, France). Endogenous peroxidase activity was blocked by treatment with 3% hydrogen peroxide in phosphate-buffered saline for 10 min. Incubation with the CD20-specific antibody was carried out at 37 °C for 30 min, and labeling was revealed using the Ventana *i*View® DAB Detection kit with copper enhancement, according to the instructions of the manufacturer. Slides were counterstained with hematoxylin and bluing reagent (Ventana). Cells were photographed using a Zeiss Axio Scope.A1 microscope and an AxioCam ICc1 camera using the Axiovision 4.8.2 software. May-Grünwald-Giemsa staining of cytological smears was done using standard methods.

### Real time PCR

Following treatments cells were centrifuged, and RNA was extracted with the RNeasy Mini Kit of Qiagen (Courtaboeuf, France). RT-PCR was performed on 1 μg of total RNA using the Iscript cDNA synthesis kit of Bio-Rad (Marne la Coquette, France) TaqMan gene expression assays were performed on an ABI prism 7000 apparatus (Applied Biosystems, Courtaboeuf, France) with the Hs 99999905_m1, Hs 0054487_m1 and Hs 01024558_m1 primer pairs (Applied Biosystems, Life Technologies, Fisher Scientific, Illkirch, France) for GAPDH, ATP2A2 (SERCA2) and ATP2A3 (SERCA3), respectively. Relative gene expression levels after treatments were calculated by the 2^-ΔΔCt^ method normalized to GAPDH, and expressed as fold change compared to untreated cells [38].

### Statistical analysis

Data are presented as the mean ± SEM, and correspond to at least three independent experiments. Statistical analysis was performed using Student’s *t* test with GraphPad Prism.

## Results

### Induction of SERCA expression in precursor B ALL cells

**Fig. 1:**
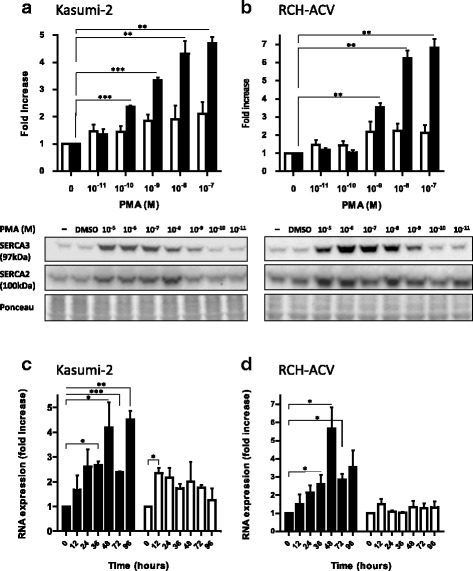
Induction of SERCA3 expression in precursor B ALL cell lines. Kasumi-2 (**a**) and RCH-ACV (**b**) cells were treated by various concentrations of PMA for 5 days, and SERCA3 (closed columns; 97 kDa) as well as SERCA2 (open columns; 100 kDa) expression was detected by Western immunoblotting (*lower part*) and quantified (*upper part*). Equal total cellular protein loading of blots was checked by Ponceau red staining. PMA treatment led to an approximately five- or sevenfold increase of SERCA3 expression in Kasumi-2 and RCH-ACV cells, respectively. Concentration-dependent induction of SERCA expression by PMA could be observed from 10^−10^-10^−9^ M and reached a plateau around 10^−8^-10^−7^ M, whereas DMSO vehicle was without effect. **c** and **d**: Kasumi-2 and RCH-ACV cells were treated with 10^−8^ M PMA, and SERCA3 (closed columns), as well as SERCA2 mRNA levels (open columns) were measured by quantitative RT-PCR and normalized to GAPDH as described in Methods. When compared to untreated cells, SERCA3 mRNA expression peaked at day 2 of treatment, and a rebound could be observed at day 4

As investigated in the Kasumi-2 and RCH-ACV cell lines that carry the t(1;19)(q23;p13) translocation and express the E2A-PBX1 fusion oncoprotein, PMA treatment led to enhanced SERCA3 expression. This could be observed from 10^−10^-10^−9^ M PMA, and reached a plateau in the 10^−8^-10^−7^ concentration range (Fig. 1a and b). Induction of SERCA3 expression was also manifested on the mRNA level. As shown in Fig. 1c and d, induction of SERCA3 mRNA expression was observed in both Kasumi-2 and RCH-ACV cells, already at 12 h of treatments, and this followed a reproducible biphasic pattern with a 5-6-fold enhancement when compared to untreated control. Moreover, the moderate enhancement of SERCA2 protein expression observed in Kasumi-2 cells could also be observed on the mRNA level.

**Fig. 2:**
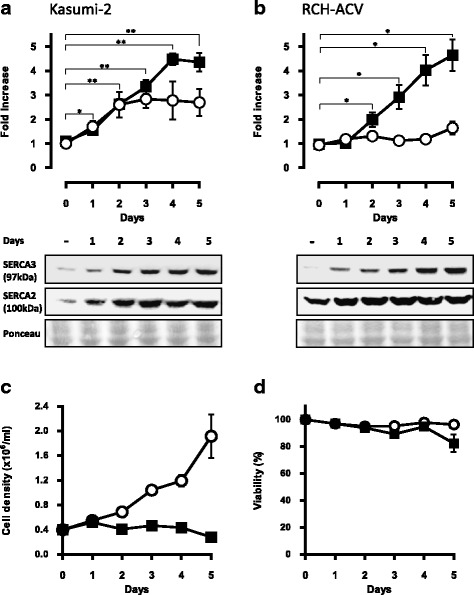
Time course of SERCA expression in PMA-treated precursor B ALL cell lines. Kasumi-2 (**a**) and RCH-ACV (**b**) cells were treated with 10^−7^ M PMA and SERCA3 (closed *squares*) as well as SERCA2 (open *circles*) expression was detected by Western blotting (*lower part*) and quantified (*upper part*). Equal protein loading was checked by Ponceau red staining of the membranes. Induction of SERCA3 expression was detected from days 1-2 and reached a plateau after 4-5 days. Treatment (Kasumi-2 cells, PMA: 10^−8^ M) led to rapid growth arrest (**c**) with maintained viability (**d**) during 5 days

Significant induction of SERCA3 protein expression was observed starting at day 1-2 and reached a plateau at day 4-5 (Fig. 2a and b). Maximal induction in the cell lines was approximately five- to sevenfold when compared to untreated cells. DMSO vehicle when applied alone was without effect. As shown in Fig. 2c and d, PMA treatment in Kasumi-2 cells led to inhibition of cell proliferation (Panel C) with maintained viability (Panel D) up till day 5, whereas longer treatments led to significant cell death.

**Fig. 3:**
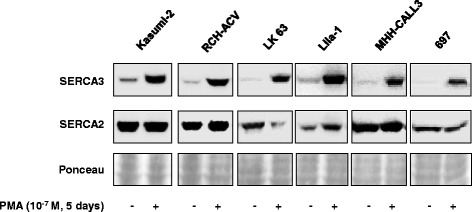
SERCA protein expression in various precursor B ALL cell lines. Induction of SERCA3 expression by PMA. Semi-quantitative Western blotting analysis of SERCA2 and SERCA3 levels in equal amounts of total cellular protein lysates obtained from untreated (-) and PMA-treated (+) cells. PMA treatment led to enhanced SERCA3 expression in all t(1;19)/E2A-PBX1^+^ cell lines

SERCA3 expression was induced by PMA in all available precursor B ALL cell lines bearing the t(1;19)/E2A-PBX1 translocation. As shown in Fig. 3, the Kasumi-2, RCH-ACV, LK63, Lila-1, MHH-CALL3 and 697 cell lines all simultaneously expressed SERCA2 and SERCA3 calcium pump isoforms. When the cells were treated with 10^−7^ M PMA for 5 days, the expression of SERCA3 protein was strongly enhanced in all cell lines, whereas expression of the SERCA2 protein remained unchanged or was slightly modified in a cell-line dependent manner. This indicates that the induction of SERCA3 expression is a universal phenomenon in all E2A-PBX1 expressing cell lines investigated in this study. On the other hand, we found that PMA treatment of precursor B ALL cell lines (Nalm-21, TOM-1, BV173, KOPN-8 and Reh) of other molecular types (see Additional file 1: Table S1) led to the death of cultures within 24–48 h.

### PMA treatment leads to cell differentiation

**Fig. 4:**
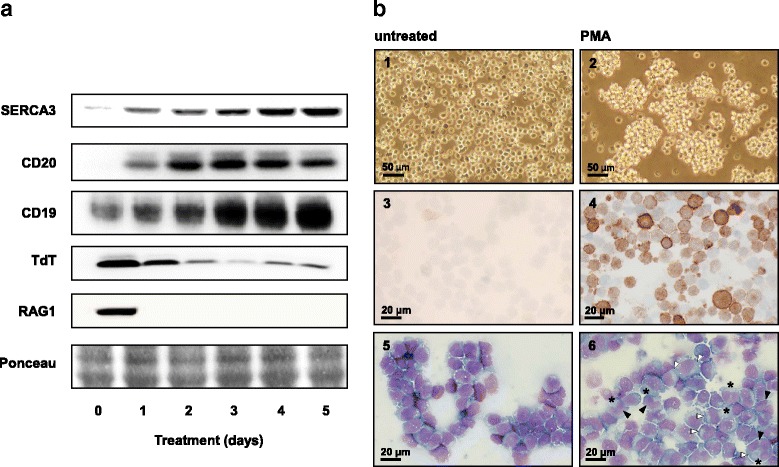
Induction of differentiation by PMA in Kasumi-2 cells. **a**: Cells were treated with 10^−7^ M PMA during 5 days and the expression of SERCA3, CD20, TdT, RAG-1 and CD19 was determined by Western blotting daily. Whereas CD19 is expressed in untreated cells, induction of SERCA3 and CD20 expression by PMA treatment was accompanied by the down-regulation of TdT and RAG-1. **b**, photographs 1 and 2: PMA treatments led to the formation of multicellular aggregates. Whereas untreated cells (Photograph 1) grew predominantly as single cells in suspension, PMA treated cells (10^−8^ M, 4 days) formed floating multicellular aggregates (Photograph 2; original magnification: 20x; bars: 50 μm). **b**, photographs 3 and 4: Induction of CD20 expression in PMA-treated Kasumi-2 cells detected by immunohistochemistry. Cytological smears of untreated (Photograph 3) and PMA-treated cells (10^−8^ M, 4 days, photograph 4) were stained for CD20 expression by immunohistochemistry as described in Methods. *Brown* staining indicates CD20 expression (original magnification: 40x; bars: 20 μm). **b**, photographs 5 and 6: May-Grünwald-Giemsa staining of cytological smears of untreated (photograph 5) and PMA-treated cells (10^−8^ M, 4 days, photograph 6). PMA treatment led greater cell size, decreased nucleocytoplasmic ratio (*asterisks*) and the frequent formation of bilobed, often excentral nuclei (*black arrowheads*) with a paranuclear halo (*white arrowheads*; original magnification: 40x; bars: 20 μm)

The phenotype of Kasumi-2 precursor B ALL cells was investigated before and after stimulation with 10^−7^M PMA for 5 days using established markers of early B cell differentiation. Whereas before treatment cells expressed high levels of TdT and RAG-1 and low levels of CD20 proteins, PMA treatment led to strong CD20 expression and to the simultaneous down-regulation of TdT and RAG-1 expression (Fig. 4a).

PMA-treatment of cells led to the formation of multicellular aggregates and to morphological changes similar to a plasmacytoid morphology (Fig. 4b). Whereas untreated cells were round, small and displayed high nucleocytoplasmic ratio and round nuclei, treatment with 10^−8^ M PMA for 4 days led to increased cell size, more abundant, often excentral cytoplasm, lower nucleocytoplasmic ratio and frequently bilobed nuclei with clear paranuclear areas.

**Fig. 5:**
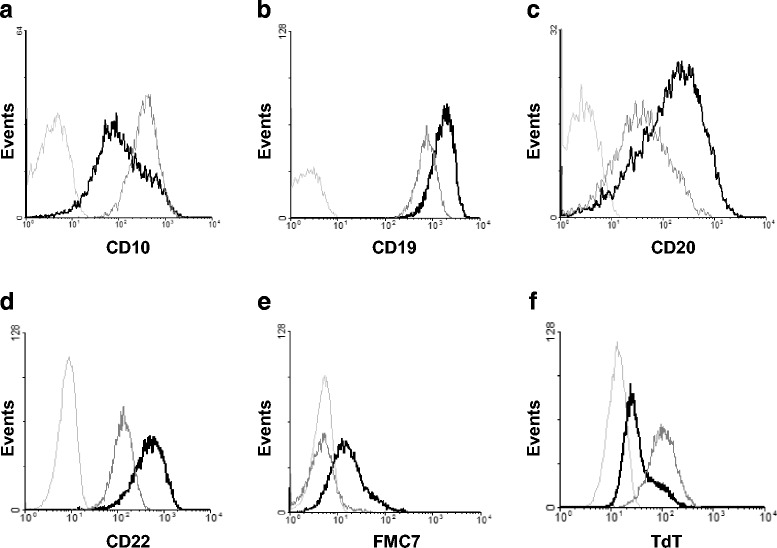
Flow cytometric analysis of phenotypic changes induced by PMA treatment. Kasumi-2 cells were treated with 10^−8^ M PMA for 4 days (*black curves*) and the expression of various early B lymphoid phenotypic markers was compared to untreated cells (*grey curves*). Appropriate isotype-matched irrelevant antibodies were used as negative control (*light grey curves*). Treatment led to increased expression of the CD19, CD20, CD22 and FMC7 differentiation markers (**b**, **c**, **d** and **e**, respectively), whereas the expression of the CD10 (**a**) and of intracellular TdT (**f**) decreased

As studied by flow cytometry, and similarly to previous observations made on the LK63 and Lila-1 t(1;19) cell lines [39], untreated Kasumi-2 cells displayed a precursor B lymphoid phenotype (CD3^−^, CD5^−^, CD10^+^, CD19^+^, CD20^+/-^, CD22^+^, CD34^−^, CD38^+^, CD45^+^, TdT^+^). As shown in Fig. 5, treatment of cells with 10^−8^ M PMA for 4 days led to markedly enhanced expression of the CD19, CD20, CD22 and FMC7 differentiation markers, whereas the expression of markers of immaturity such as CD10 or TdT was decreased. PMA treatment, however, did not lead to κ or λ chain or IgM expression (not shown), indicating however, that the cells do not attain a mature B cell phenotype.

### Induction of SERCA3 expression is PKC-dependent

**Fig. 6:**
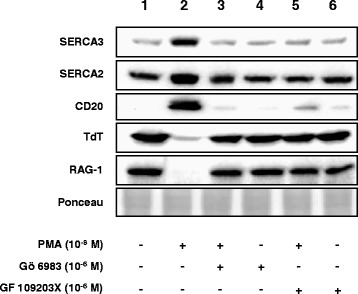
Effect of PKC inhibitors on PMA-induced cell differentiation. Kasumi-2 cells were treated with 10^−8^ M PMA in the presence or the absence of the Gö 6983 or the GF 109203X PKC inhibitor for 4 days, and the expression of SERCA3 and SERCA2, as well as of CD20, TdT and RAG-1 proteins was detected by Western blotting as described in Methods. Equal loading was controlled by Ponceau red staining. When compared to untreated cells (lane 1), PMA treatment alone (lane 2) led to the induction of SERCA3 and CD20 expression and the down-regulation of TdT and RAG-1 proteins. Gö 6983 or GF 109203X abolished the effects of PMA (lanes 3 and 4). When applied alone, Gö 6983 or GF 109203X had no significant effect of the cells (lanes 5 and 6)

The induction of SERCA3 expression by PMA was investigated in the presence or absence of pharmacological inhibitors of PKC. As shown in Fig. 6, when cells were preincubated with Gö 6983 or with GF 109203X for 30 min, the consecutive addition of 10^−8^ M PMA (a maximally active concentration) was without effect on SERCA expression, indicating the involvement of conventional or novel-type PKC activity in PMA-induced SERCA expression.

### Comparison of SERCA3 expression in precursor B and mature B cell lines

**Fig. 7:**
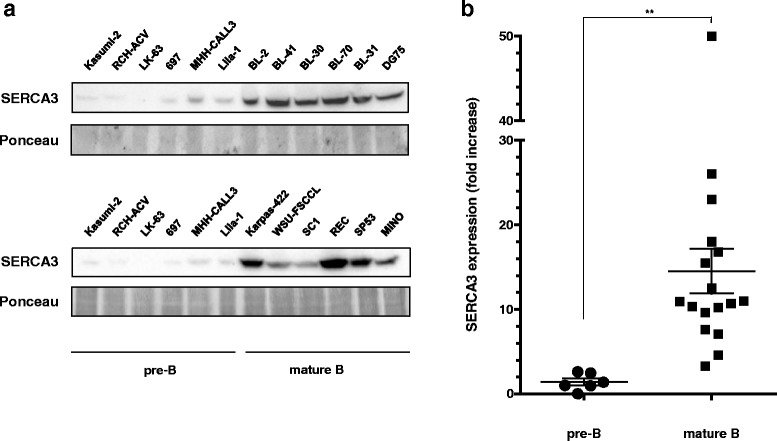
SERCA3 expression levels in precursor B and mature B cell lines. **a**: SERCA3 expression was determined by semi-quantitative Western blotting using total protein lysates prepared from various untreated precursor-B and mature B cell lines (see Additional file 1: Table S1). Protein loading was checked by Ponceau red staining. **b**: SERCA3 levels are expressed as fold change compared to untreated Kasumi-2 cells. Mature B cells express significantly higher levels of SERCA3. When SERCA3 expression is compared between pre-B and mature B cells as a group, an approximately fifteen fold higher expression is observed in mature B cells in a highly significant manner (*p* = 0.0087)

In order to further correlate SERCA3 expression with the state of differentiation of neoplastic B cells, SERCA3 expression of precursor B cell lines was compared to that of an extensive set of randomly selected mature B cell lines (see Additional file 1: Table S1). As shown in Fig. 7, SERCA3 expression in all individual precursor B cell lines was weaker than in any of the investigated mature cell lines. A highly significant, on average 15-fold higher SERCA3 expression could be observed in mature B cell lines when compared with the average expression in the precursor B cell group.

### SERCA activity is involved in the control of cell differentiation

**Fig. 8:**
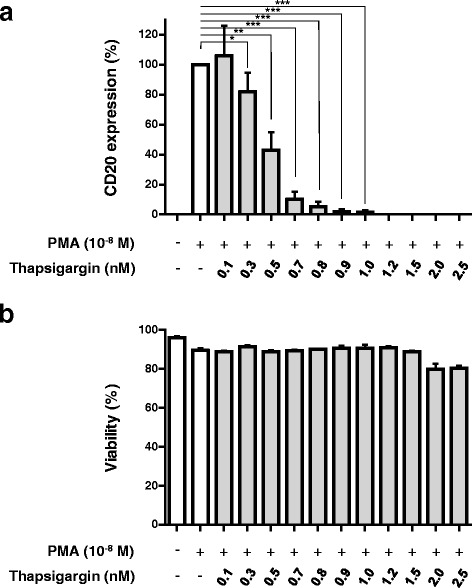
Effect of SERCA inhibition on PMA-induced expression of CD20 in Kasumi-2 cells. **a**: Cells were incubated with 10^−8^ M PMA in the absence (*empty column*) or in the presence (*gray columns*) of various thapsigargin concentrations in the sub- to low nanomolar concentration range for 4 days, and CD20 expression was measured by semi-quantitative Western blotting. Thapsigargin treatment led to a concentration-dependent inhibition of PMA-induced CD20 expression. Although higher concentrations of thapsigargin led to cell death, no major cytotoxic effects were observed at the concentrations used in these experiments (**b**)

The role of SERCA function in the differentiation of leukemic B cells was investigated using thapsigargin, a high affinity, selective inhibitor of SERCA-dependent calcium transport [40]. As shown in Fig. 8, Treatment of Kasumi-2 cells with sub-nanomolar concentrations of thapsigargin completely abolished the induction of CD20 expression induced by PMA (Fig. 8a). Inhibition of PMA-induced CD20 expression by thapsigargin was observed in the absence of significant toxicity during the experiments (Fig. 8b), although higher thapsigargin concentrations were, in accordance with the literature [41], toxic. Similar observations were made with cyclopiazonic acid or 2,5-di-*tert*-butyl-1,4-benzohydroquinone, two other, structurally unrelated SERCA inhibitors as well (data not shown).

## Discussion

We show for the first time that ER calcium pump expression is remodeled during early B lymphoid differentiation. Activation of PKC-dependent signaling with a phorbol ester-type DAG analog in the Kasumi-2 B ALL cell line, blocked at the precursor B stage, led to the induction of differentiation of the cells as detected by the loss or decrease of expression of CD10, TdT and RAG-1 and the induction of expression of the CD19, CD20, CD22 and FMC7 markers, as well as morphological changes. This process was accompanied by a marked induction of the expression of SERCA3 mRNA and protein, whereas the expression of SERCA2 was only moderately increased. SERCA3 induction was observed on the protein, as well as the mRNA level. Whereas SERCA3 protein induction reached a plateau, mRNA induction followed a biphasic pattern. This may be due the dynamic interplay of various regulatory mechanisms, these, however, remain to be identified.

The induction of SERCA3 expression upon PMA treatment was observed in all B ALL cell lines investigated that carry the t(1;19) translocation giving rise to the E2A-PBX1 fusion oncoprotein, whereas in cell lines that belong to other molecular types such as t(9;22), t(11;19) or t(12;21) that express the BCR-ABL, MLL-ENL or TEL-AML1 fusion oncoproteins, respectively, we observed that PMA treatment in the same conditions led to rapid cell death. This suggests that the E2A-PBX1 expressing B ALL may constitute a molecular subtype in which differentiation blockage can be overcome by PKC activation. This may help to better understand the molecular mechanisms responsible for the inhibition of differentiation of this type of leukemia, should be investigated on primary leukemic cells ex vivo, and may be of potential importance for the development of targeted therapies in the future.

In addition to activating protein kinase C (PKC) isoenzymes belonging to the conventional and novel subfamilies, PMA can bind to and activate other DAG-binding proteins [42–47], including the RasGRP1 guanine nucleotide exchange factor [48]. However, the inhibition of the effects of PMA on the expression of SERCA3 and of other differentiation markers by PKC inhibitors indicates that PKC activation is the essential initiating step of the pharmacologically induced differentiation process that leads to the induction of SERCA3 expression in E2A-PBX1-expressing t(1;19) precursor B ALL cells. The precise identification of the PKC isoforms involved will require further studies such as, for example, PKC isoform-specific knock-down or gene invalidation.

Strong SERCA3 expression has already been observed in normal mature resting B lymphocytes *in situ* in lymph node follicles [20], and an approximately fivefold increase of SERCA3 mRNA levels can be seen in the ImmGen database [49] when pro- and pre-B cell populations and newly formed B cells are compared (see Additional file 2: Figure S1 and and Additional file 3 Figure: S2 for the expression profile of SERCA3, SERCA2 and various established differentiation markers, respectively), suggesting that enhanced SERCA3 expression occurs during normal B lymphocyte differentiation. The investigation of the cross-talk between key B cell differentiation regulatory mechanisms and SERCA function during in vitro differentiation of normal B lymphocyte precursors will help to better understand the underlying regulatory mechanisms, as well as the role of ER calcium homeostasis in B lymphocyte maturation.

Our work indicates that E2A-PBX1-transformed B-ALL cells can be induced to differentiate by PKC activation, and that induction of SERCA3 mRNA and protein expression is induced during this process. A sharp contrast could also be observed when SERCA3 expression of precursor B ALL cell lines was compared to a large set of cell lines encompassing a wide range of mature B neoplasia types. Despite variation among different cell lines, SERCA3 expression was several-fold higher in mature cells when compared to precursor B cell lines. This indicates that systematic differences exist in terms of SERCA3 expression between immature and mature B lymphoid malignancies, and that SERCA3 may therefore constitute a useful new phenotypic marker for the study of B lymphoid differentiation and leukemia/lymphoma phenotype analysis.

Calcium signaling plays an important role in the activation of mature B cells [5, 50]. Antigen binding to the BCR leads to phospholipase-C activation, IP3 production and calcium release from IP3-sensitive intracellular pools [50–52]. The calcium affinity of SERCA3 is inferior when compared to the simultaneously expressed SERCA2 isoform [21; 23], and SERCA3 has been shown to be preferentially associated with the IP3-sensitive intracellular calcium compartment in platelets [53]. It is therefore tempting to hypothesize that the induction of SERCA3 expression during the maturation of B cell precursors is part of the differentiation program, whereby the cells acquire an intracellular calcium storage compartment poised to mount larger calcium release signals upon BCR activation.

When cell differentiation was conducted in the presence of sub-nanomolar, non-toxic concentrations of thapsigargin, a high affinity *pan*-SERCA inhibitor, induction of CD20 expression by PMA was completely suppressed. Similar results were obtained by using cyclopiazonic acid and 2,5-di(*tert*)butyl-1,4-benzohidroquinone, two other, structurally unrelated SERCA inhibitors as well. These observations show, that SERCA function is required for differentiation to proceed, and that the perturbation of SERCA activity can interfere with this process. The identification of the relative contribution of SERCA3 and SERCA2 to cell differentiation will require further studies based on the direct, selective modulation of SERCA3 and SERCA2 levels. This can be accomplished by isoenzyme-specific transgene overexpression, gene invalidation or knock-down techniques. When applied in conjunction with genetically engineered fluorescent calcium indicators (GECIs) targeted to the ER lumen or the cytosol, these studies will allow to better understand the functional cross-talk that occurs between ER calcium homeostasis and the control of cell differentiation and its defects in leukemia.

## Conclusions

In summary, this work shows, for the first time that SERCA3 expression is induced during early B cell differentiation, inhibition of SERCA3 expression and the differentiation block in pre-B ALL cells can be overcome by PKC activation, and that unperturbed SERCA function is required for cell differentiation. In addition, these data indicate that SERCA3 may serve as a useful new phenotypic marker for the study of B cell differentiation and its defects in leukemia.

## Additional files

Additional file 1: Table S1. Name, hematological origin and molecular type of the cell lines used in this study. (DOCX 18 kb)

Additional file 2: Figure S1. Expression profile of SERCA3 and SERCA2 mRNA in key normal early B cell populations in the mouse, adapted from the Immunological Genome project transcriptomic database. (PPTX 1190 kb)

Additional file 3: Figure S2. Expression profile of CD19, CD20 CD22, CD34, TdT and of β-actin in key normal early B cell populations in the mouse, adapted from the Immunological Genome project transcriptomic database. (PPTX 566 kb)

## Abbreviations

ALL: acute lymphoblastic leukemia
ATCC: American Type Culture Collection
BCR: B cell antigen receptor
DAB: 3,3’-diaminobenzidine
DAG: diacyl-glycerol
DMSO: dimethyl-sulfoxide
DSMZ: Deutsche Sammlung von Mikroorganismen und Zellkulturen GmbH
E2A: transcription factor 3 (TCF3)
EBV: Epstein-Barr virus
ECACC: European Collection of Cell Cultures
ER: endoplasmic reticulum
GAPDH: glyceraldehyde 3-phosphate dehydrogenase
IP3: D-*myo*-inositol-1,4,5-trisphosphate
NFAT: Nuclear factor of activated T cells
NF-κB: Nuclear Factor kappa-light-chain-enhancer of activated B cells
PBX1: pre-B-cell leukemia transcription factor 1
PKC: protein kinase C
PLC-γ: phospholipase C-gamma
PMA: phorbol-12-myristate-13-acetate
RAG-1: recombination-activating gene 1
RT-PCR: reverse transcription polymerase chain reaction
SDS PAGE: Sodium dodecyl-sulfate polyacrylamide gel electrophoresis
SERCA: sarco/endoplasmic reticulum calcium ATPase
TCA: trichloroacetic acid
TdT: Terminal deoxynucleotidyl transferase
