# Supplementary material for: Induction of endoplasmic reticulum calcium pump expression during early leukemic B cell differentiation
Source: J Exp Clin Cancer Res. 2017 Jun 26;36:87. doi: 10.1186/s13046-017-0556-7 (PMC5485704; doi:10.1186/s13046-017-0556-7)
Supplement: Supplementary file 1 — Name, hematological origin and molecular type of the cell lines used in this study. (DOCX 18 kb) [file 13046_2017_556_MOESM1_ESM.docx]

**Table S1 : Origin and characterization of the cell lines used in this study**

| **Cell line** | **Hematological origin** | | **Reference** |
| --- | --- | --- | --- |
| **Kasumi-2** | *Precursor B ALL cell lines* | t(1;19) E2A-PBX1 | Akbari Moqadam et al. *Leukemia* 2014^#^ |
| **RCH-ACV** |  |  | Jack et al. *Cancer Genet Cytogenet* 1986^#^ |
| **LK-63** |  |  | Salvaris et al. *Leuk Res* 1992 |
| **Lila-1** |  |  |  |
| **MHH-CALL-3** |  |  | Tomeczkowski et al. *Br J Haematol* 1995^#^ |
| **697** |  |  | Findley et al. *Blood* 1982^#^ |
| **Nalm-21** |  | t(9;22) BCR-ABL | Matsuo et al. *Hum Cell* (1991)^#^ |
| **TOM-1** |  |  | Okabe et al. *Blood* 1987 |
| **BV173** |  |  | Pegoraro et al. *J Natl Canc Inst* 1983 |
| **KOPN-8** |  | t(11;19) MLL-ENL (MLL-MLLT1) | Nakazawa et al. *Jpn J Clin Hematol* 1978^#^ |
| **Reh** |  | t(12;21) TEL-AML1 (ETV6-RUNX1) | Rosenfeld et al. *Nature* 1977^#^ |
| **BL-2** | *Mature B cell lines* | Burkitt’s lymphoma (EBV^neg^) | Lenoir et al. *IARC Sci Publ* 1985 |
| **BL-30** |  |  |  |
| **BL-31** |  |  |  |
| **BL-41** |  |  |  |
| **BL-70** |  |  |  |
| **D.G.-75** |  |  | Ben-Bassat et al. *Intl J Cancer* 1997 |
| **BJA-B** |  |  | Menezes et al. *Biomedicine* 1975 |
| **Ramos** |  |  | Klein et al. *Intervirology* 1975* |
| **HBL-2** |  | mantle cell lymphoma | Abe et al. *Cancer* 1988 |
| **MAVER-1** |  |  | Zamo et al. *Haematologica* 2006 |
| **JEKO-1** |  |  | Jeon et al. *Br. J. Haematol.* 1998 |
| **Mino** |  |  | Lai et al. *Leuk Res* 2002^°^ |
| **REC-1** |  |  | Raynaud et al. *Genes Chromosomes Cancer* 1993 |
| **Karpas-422** |  | follicular lymphoma | Dyer et al. *Blood* 1990^#^ |
| **Sc-1** |  |  | Th’ng et al. *Int J Cancer* 1987^#^ |
| **WSU-FSCCL** |  |  | Mohammad et al. *Cancer Genet Cytogenet* 1993^#^ |

(^#^DSMZ, °ATCC, *ECACC).
