# Supplementary material for: Induction of endoplasmic reticulum calcium pump expression during early leukemic B cell differentiation
Source: J Exp Clin Cancer Res. 2017 Jun 26;36:87. doi: 10.1186/s13046-017-0556-7 (PMC5485704; doi:10.1186/s13046-017-0556-7)
Supplement: Supplementary file 2 — Expression profile of SERCA3 and SERCA2 mRNA in key normal early B cell populations in the mouse, adapted from the Immunological Genome project transcriptomic database. (PPTX 1190 kb) [file 13046_2017_556_MOESM2_ESM.pptx]

## Slide 1
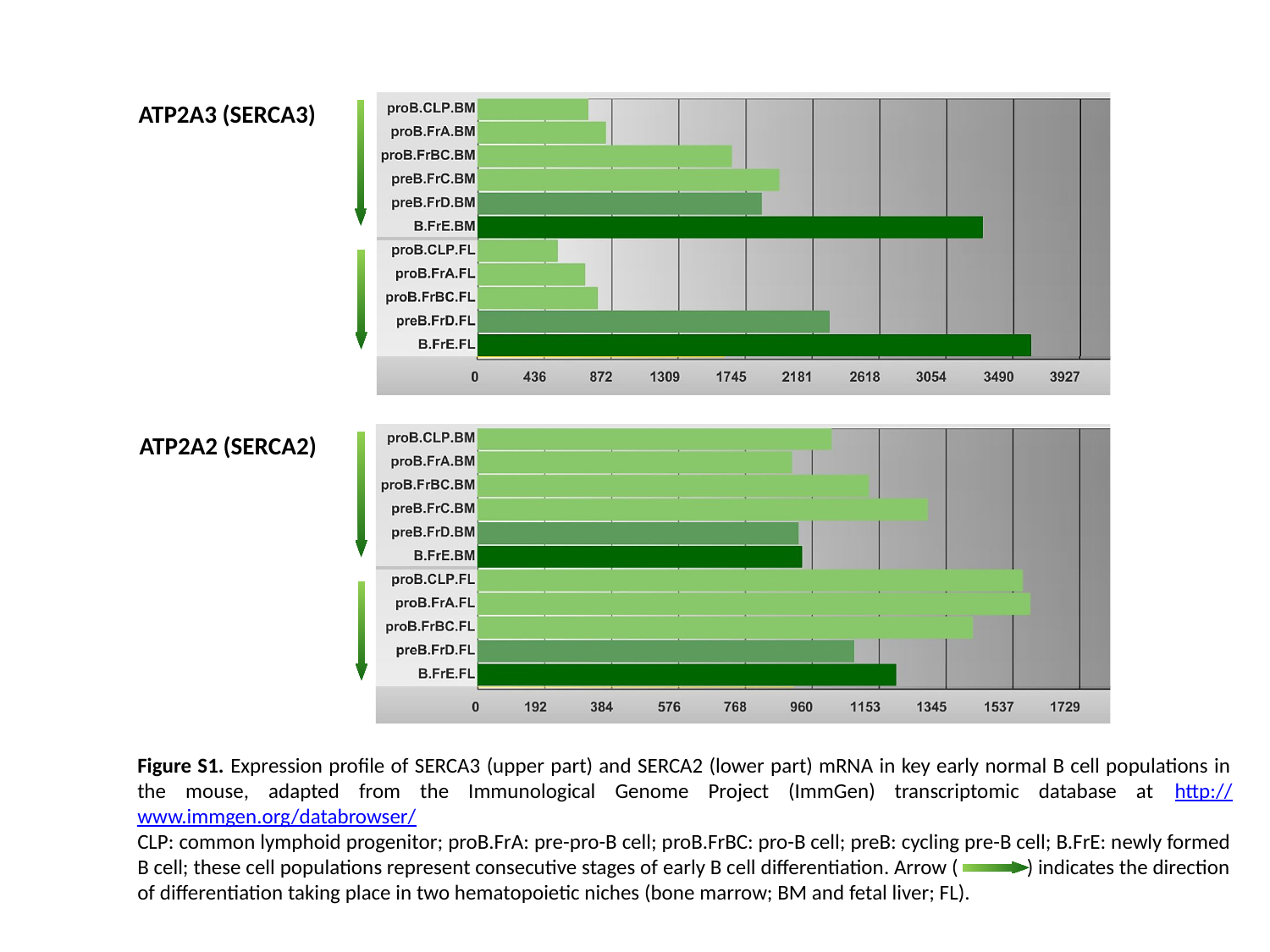

ATP2A3 (SERCA3)
ATP2A2 (SERCA2)
Figure S1. Expression profile of SERCA3 (upper part) and SERCA2 (lower part) mRNA in key early normal B cell populations in the mouse, adapted from the Immunological Genome Project (ImmGen) transcriptomic database at http://www.immgen.org/databrowser/
CLP: common lymphoid progenitor; proB.FrA: pre-pro-B cell; proB.FrBC: pro-B cell; preB: cycling pre-B cell; B.FrE: newly formed B cell; these cell populations represent consecutive stages of early B cell differentiation. Arrow ( ) indicates the direction of differentiation taking place in two hematopoietic niches (bone marrow; BM and fetal liver; FL).
