# Supplementary material for: Induction of endoplasmic reticulum calcium pump expression during early leukemic B cell differentiation
Source: J Exp Clin Cancer Res. 2017 Jun 26;36:87. doi: 10.1186/s13046-017-0556-7 (PMC5485704; doi:10.1186/s13046-017-0556-7)
Supplement: Supplementary file 3 — Expression profile of CD19, CD20 CD22, CD34, TdT and of β-actin in key normal early B cell populations in the mouse, adapted from the Immunological Genome project transcriptomic database. (PPTX 566 kb) [file 13046_2017_556_MOESM3_ESM.pptx]

## Slide 1
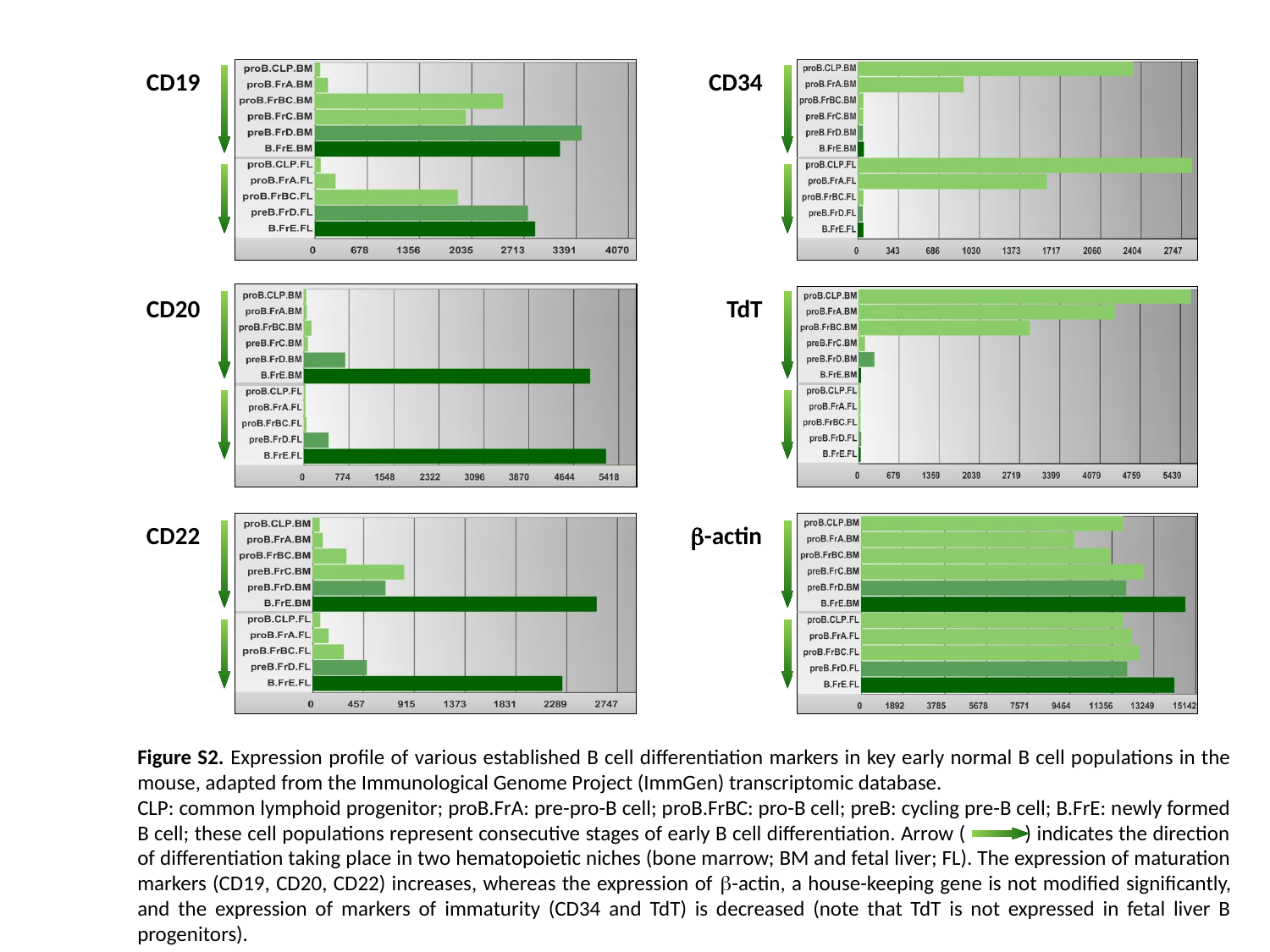

CD19
CD34
CD20
TdT
CD22
b-actin
Figure S2. Expression profile of various established B cell differentiation markers in key early normal B cell populations in the mouse, adapted from the Immunological Genome Project (ImmGen) transcriptomic database.
CLP: common lymphoid progenitor; proB.FrA: pre-pro-B cell; proB.FrBC: pro-B cell; preB: cycling pre-B cell; B.FrE: newly formed B cell; these cell populations represent consecutive stages of early B cell differentiation. Arrow ( ) indicates the direction of differentiation taking place in two hematopoietic niches (bone marrow; BM and fetal liver; FL). The expression of maturation markers (CD19, CD20, CD22) increases, whereas the expression of b-actin, a house-keeping gene is not modified significantly, and the expression of markers of immaturity (CD34 and TdT) is decreased (note that TdT is not expressed in fetal liver B progenitors).
